# Monte Carlo simulation and unfolding of an extended Bonner sphere system for secondary neutron spectrometry in proton therapy facilities using conventional BSS experimental data

**DOI:** 10.3389/fonc.2026.1789835

**Published:** 2026-04-13

**Authors:** Adrián Díaz-Comeche, Sandra Oliver, Belén Juste, Daniel Jardin, Mark Zakhary, Sina Mossahebi, Matthew M. Mille, Choonsik Lee, Rafael Miró, Gumersindo Verdú, Sergio Morató

**Affiliations:** 1 Institute for Industrial, Radiophysical and Environmental Safety, Universitat Politècnica de València, Valencia, Spain; 2 Radiation Physics Division National Institute of Standards and Technology, Gaithersburg, MD, United States; 3 Maryland Proton Treatment Center, University of Maryland School of Medicine, Baltimore, MD, United States; 4 Department of Radiation Oncology University of Florida College of Medicine, Gainesville, FL, United States; 5 University of Florida Health Proton Therapy Institute, Jacksonville, FL, United States; 6 Division of Cancer Epidemiology and Genetics, National Cancer Institute, National Institutes of Health, Rockville, MD, United States

**Keywords:** Bonner sphere spectrometer, experimental measurements, Monte Carlo simulation, proton therapy, secondary neutron radiation

## Abstract

The extended-range Bonner Sphere Spectrometer (BSS) is a valuable tool for characterizing neutron fields in radiotherapy, including proton therapy facilities, where secondary neutrons contribute to the incidental dose received by the patient and staff. In this study we developed and validated a Monte Carlo (MC) model of an extended-range BSS by benchmarking against experimental measurements performed using a conventional BSS in a proton therapy facility. The extended-range system, which includes four additional spheres with aluminum and lead layers, was not available for this work; instead, simulations were validated against conventional BSS data and then applied to extrapolate the high-energy portion of the neutron spectrum. The MC simulations were performed using MCNP version 6.3 and include modelling of the BSS and using BSS response functions previously validated. The comparison in data trends between simulations and measurements confirms the reliability of the simulation model. The acquired neutron spectra show the characteristic shape, but also noticeable differences between the measurement locations in the intensity and width of the direct, evaporation, and thermal peaks. These differences are consistent with the changing measurement conditions in each case. The obtained spectra show similarity with other spectra available in literature. This work demonstrates that MC-modeled extended-range BSS can accurately characterize secondary neutron spectra in proton therapy facilities, providing a foundation for improved radiation protection and dose optimization studies.

## 1 Introduction

Proton therapy is a highly conformal radiotherapy technique that enables precise dose delivery to tumors while reducing exposure to surrounding healthy tissue. However, interactions between the proton beam and various components of the treatment environment—including the beam delivery system (nozzle), treatment room structures, and the patient—produce secondary neutrons, which contribute to the incidental dose received by the patient and clinical staff (1). The resulting secondary neutrons span a wide energy range, from thermal energies up to the energy of the proton beam (typically 50 MeV to 250 MeV) (2–4). Because neutron dose equivalent is strongly energy-dependent (5), accurate characterization of the secondary neutron energy spectrum is essential for radiation protection, treatment optimization, and the evaluation of long-term cancer risk (6).

While numerous studies describe experimental measurements of ambient and personal neutron dose equivalent in proton therapy rooms (7–10), far fewer have directly measured the secondary neutron energy spectrum (11–13). A recent review paper by Vedelago et al. (14) summarizes the neutron dosimetry approaches and the current state of knowledge in the field. The Bonner Sphere Spectrometer (BSS) is the primary instrument used for measuring neutron energy spectra. Most conventional BSS systems consist of six high-density polyethylene moderator spheres with diameters of 5.08 cm (2 in), 7.62 cm (3 in), 12.70 cm (5 in), 20.32 cm (8 in), 25.4 cm (10 in), and 30.48 cm (12 in). These will henceforth be identified as sphere 1, 2, 3, 4, 5, and 6, respectively. The conventional BSS system has an operational energy range from thermal energies up to about 12 MeV, while extended-range systems incorporate additional spheres with high-Z materials to improve sensitivity to high-energy neutrons, which is especially relevant for proton therapy environments (12, 15, 16). However, extended-range BSS systems are expensive, often custom-built, and are available at only a few research centers, limiting their widespread use.

Monte Carlo (MC) simulations offer a convenient alternative approach to physical measurements and are commonly used for detector modelling and shielding design applications (17–19). Simulations offer greater flexibility and can provide detailed insights that are sometimes not achievable experimentally, but they depend on accurate input data and modelling assumptions. Conversely, experimental methods can provide direct validation, but are limited by detector response, resource demands, and practical constraints. As a result, most studies tend to rely exclusively on simulations (13, 20–22) or measurements (10, 12, 23), with only a few combining both approaches (11, 17). However, neutron generation strongly depends on the treatment room design (13), highlighting the need for dedicated measurements and simulations in different facilities. In particular, few studies using extended-range BSS address Pencil Beam Scanning proton therapy systems, and to date no work combining extended-range BSS measurements with simulations has been reported for a Varian ProBeam accelerator.

In this study, we developed a MC model of an extended-range BSS to unfold the secondary neutron spectrum in a proton therapy treatment room with Varian ProBeam PBS system. The model was based on an extended-range BSS detector design described by (24, 25) which consists of four additional spheres with layers of aluminum and lead that will be referred to as sphere 7, 8, 9, and 10. Because the physical extended-range spheres were not available for this study, we benchmarked the MC simulations against experimental measurements using a conventional BSS system. However, additional simulations of the extended-range BSS spheres are included to provide a framework for comparing future measurements once an extended-range BSS becomes available.

## 2 Materials and methods

**Figure 1:**
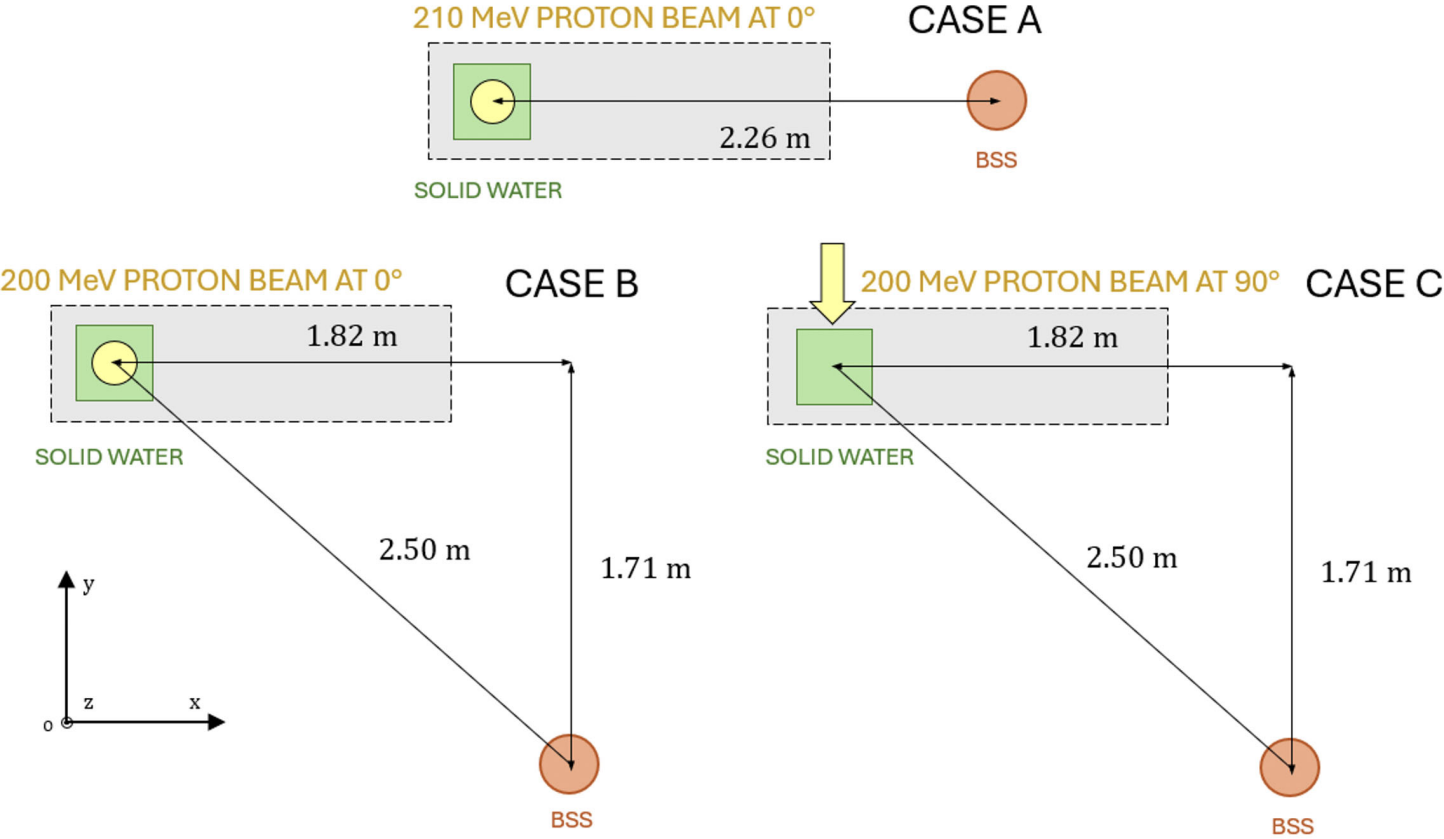
Illustration from above of the relative positions of the Solid Water block (green), beamline (yellow), couch (gray), and detector (red) for each measurement. The x-axis is parallel to the rotational axis of the Varian ProBeam, the y-axis is perpendicular to that in the plane of the gantry rotation, and the z-axis is vertical when standing in the room. Cases A (top) and B (left) correspond to vertical irradiation onto the top of the block, and Case C (right) involves lateral irradiation from the right side. The beam travels towards negative z in cases A and B, and negative y in case C.

**Figure 2:**
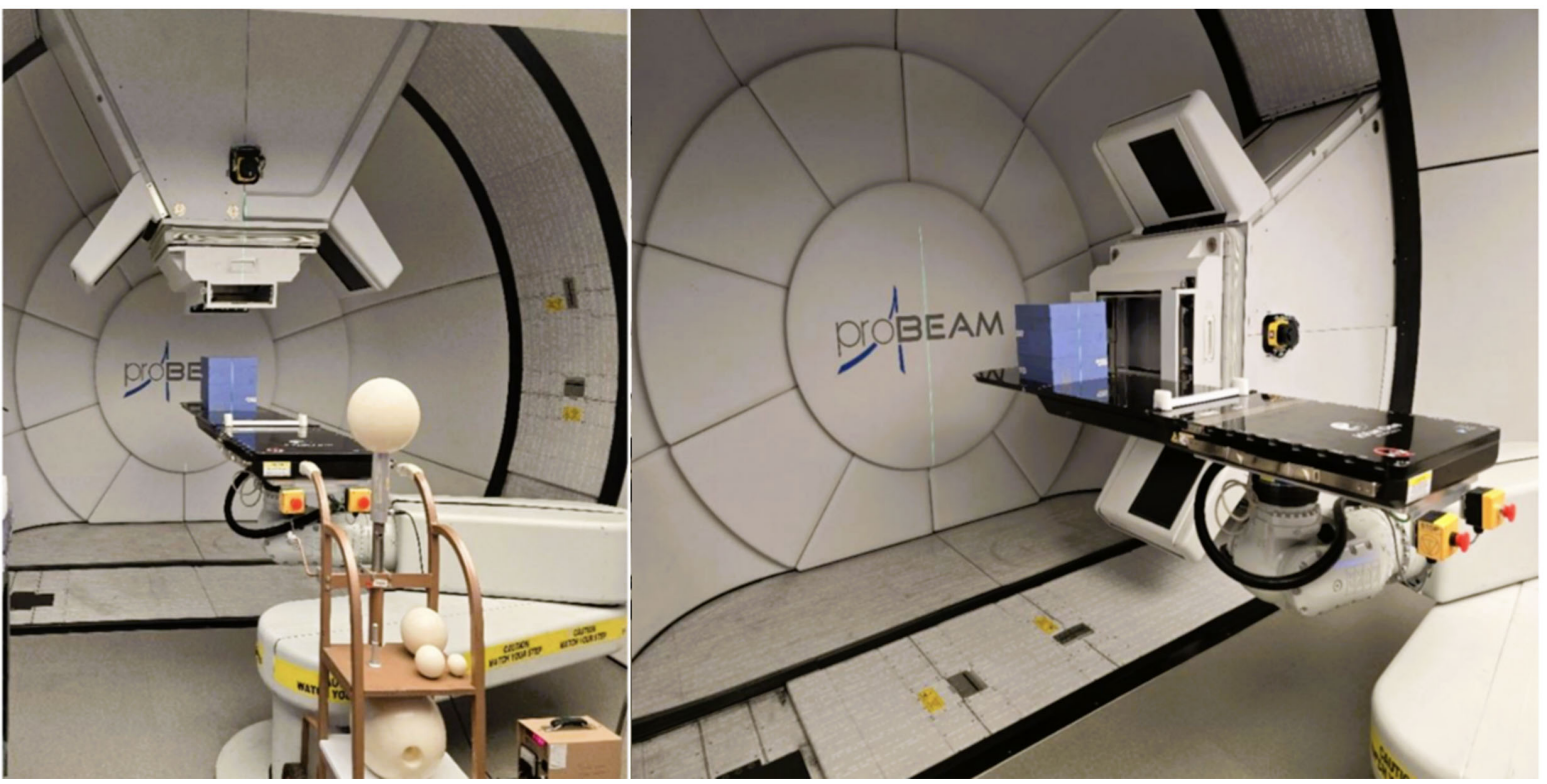
Photographs of the experimental setup at the MPTC showing the BSS measurement setup for Cases A and B with the gantry oriented at 0° (left), with gantry oriented at 90° (right) for Case C. The blue Solid Water phantom can be seen on the treatment table in both images.

Measurements were performed using a Varian ProBeam (Varian Medical Systems, Palo Alto, CA) pencil beam scanning proton therapy system located at the Maryland Proton Treatment Center (MPTC) in Baltimore, Maryland, USA. More information related to the cyclotron, nozzle, and gantry components of the accelerator at the MPTC can be found in a previous publication (26). The experimental setup consisted of a monoenergetic proton beam directed at a Solid Water High Equivalency (HE) block (27) with dimensions 30 cm × 30 cm × 30 cm serving as a tissue-equivalent medium for secondary neutron production that scatters through the treatment room. Three detector–gantry configurations were considered in this study (Cases A, B, and C). Case A uses a monoenergetic beam of 210 MeV, while Cases B and C use a 200 MeV beam. The gantry angle is 0° (straight down) in Cases A and B, and 90° in Case C. **Figure 1** provides a schematic representation of the detector positions relative to the Solid Water block and the beamline orientations for the three cases. Photographs of the experimental setups are shown in **Figure 2**.

### 2.1 Experimental BSS measurements

A conventional BSS (28) was used to detect the generated neutrons at the MPTC facility. A Ludlum Model 42–5 neutron ball cart detector (Ludlum Measurements Inc., Sweetwater, TX) (29) was used and has an operational range from thermal neutrons up to 12 MeV. The detector was previously calibrated with a certified, NIST-traceable 4.9 Ci Am-241 source. The spectral information comes from the use of spheres of different sizes to vary the degree of neutron moderation. The conventional BSS consists of a set of six moderator spheres of high-density polyethylene with density of 0.95 g cm^-3^. The detector is a cylindrical scintillator crystal made of ^6^LiI(Eu) with a volume of 0.050 cm^3^ (measuring 0.4 cm in both length and diameter) and a density of 3.494 g cm^-3^ (30). A thermal neutron detector placed at the center of each sphere counts the moderated neutrons, and the incident neutron energy spectrum can be reconstructed by using multiple spheres. In each of the three experiments, the neutron counts were measured using all six spheres. The delivered monitor units (MU) were kept constant for Cases B and C at 35–141 MU for each measurement, while for Case A about 295–000 MU were delivered. For the experimental neutron counts, an uncertainty of 10% has been considered, according to the detector manual.

### 2.2 Monte Carlo simulations

**Figure 3:**
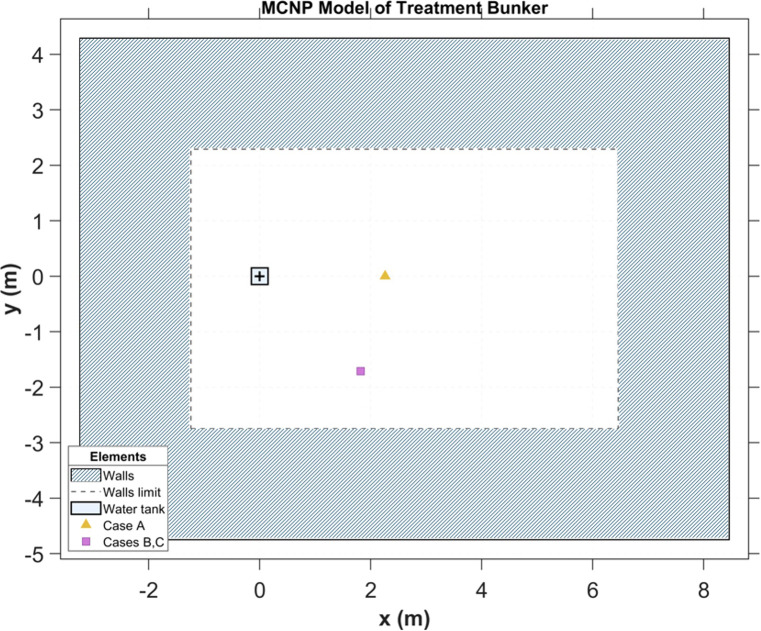
Top view of the MCNP model of the treatment bunker. The water tank (cyan) is positioned 1.10 m from the left concrete wall. Case A (gold) is positioned at (x,y) = (+2.26 m,0 m) and Cases B and C (magenta) at (x,y) = (+1.82 m,-1.71 m) relative to the tank center. The 7.70 m × 5.04 m treatment area is enclosed by concrete walls 2 m thick. All coordinates are relative to the center of the water tank.

MC simulations were performed using the MCNP 6.3 (31), a general-purpose MC radiation transport code. The simulations included a Solid Water block and a monoenergetic point source positioned 100 cm from its surface. A simple model of the MPTC treatment room bunker walls was included as it is important to account for neutrons that collide with the bunker components and return to the detector (32, 33). All walls were modelled as 2 m thick and composed of concrete with a density of 2.30 g cm^-^³, based on the NIST Portland formulation (34), which has also been used in previous work (11). A top view diagram of the MCNP model of the treatment bunker and the measurement positions is shown in **Figure 3**.

#### 2.2.1 Simulated BSS measurements

The neutron particle fluence per history was registered by a F4 tally (units of cm^-2^) placed at the same position as the BSS in the experiments for Cases A, B, and C. The secondary neutron energy-fluence spectrum was calculated using 114 logarithmically spaced energy bins ranging from 0.001 eV to 250 MeV. A sufficient number of particle histories (approximately 2×10^10^) were run to ensure the MC statistical uncertainties were below 2.5% for all tally bins.

The response of the extended-range BSS is defined as the expected number of neutron counts per unit of fluence. Hence, to obtain the neutron counts on the detector, each component of the neutron fluence energy spectrum was multiplied by each sphere’s response at that energy. The total number of counts with each sphere was calculated as:


$$m_{i}=\sum_{j=1}^{n}R_{ij}\emptyset_{j},$$

where $m_{i}$ is the detector neutron counts for the *i*-th sphere, *n* is the number of energy points composing the response matrix, ∅ is the neutron energy fluence spectrum obtained with the F4 tally and *R* is the response matrix of the extended-range BSS in units of cm^2^. An extended-range BSS response matrix was developed through Monte Carlo simulations as described in a previous publication by 35. The total uncertainty in the simulated neutron counts accounts for the statistical uncertainty of the fluence tally as well as the uncertainty in the BSS response matrix.

All defined materials in the MCNP simulations used the continuous-energy cross sections available for protons and neutrons in the standard package taken from Evaluated Nuclear Data Files (ENDF/B-VII) (36). The polyethylene and aluminum in the extended-range spheres include the neutron thermal scattering data S(α,β) from the ENDF/B-VII.1 to describe the effects due to chemical binding. The simulation of neutron interactions at energies higher than 20 MeV required the use of intra-nuclear cascade models and evaporation models, due to the lack of cross section libraries over that energy. In this work the intra-nuclear cascade model CEM03.03 and the evaporation model GEM were used, following the approach of previous studies (12, 37).

#### 2.2.2 Beam validation

To validate our MC proton beam model, we compared measured and calculated depth-dose curves for monoenergetic proton beams incident in a liquid water tank (dimensions 30 cm × 30 cm × 30 cm). The position of 90%, 80%, and 20% dose levels in the distal falloff (R90, R80, and R20) were measured with a PTW Bragg Peak Chamber Type 34070 (PTW‐Freiburg, Germany) which has a nominal sensitive volume of 10.5 cm^3^ and water-equivalent thickness (WET) of 4 mm (26). The values of R90, R80, and R20 were corrected for the WET of the utilized chamber. Experiments were conducted with 200 MeV and 210 MeV monoenergetic proton beams, as high treatment energies produce the most secondary neutrons (11).

**Figure 4:**
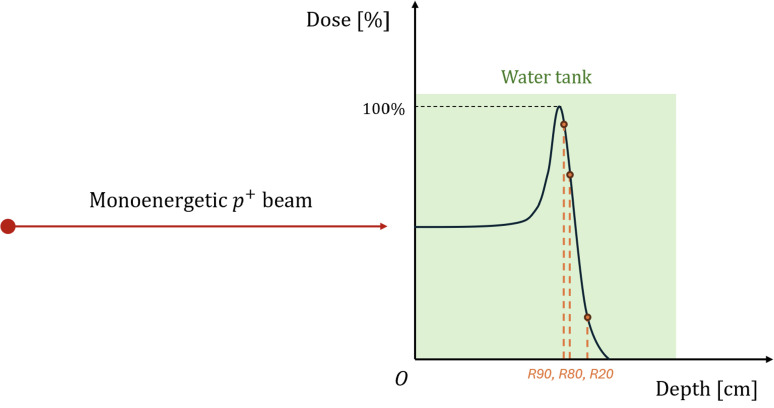
Schematic representation of the MC model used to calculate the depth–dose curves in the water tank. The points R90, R80, and R20 correspond to the depths at which the absorbed dose falls to 90%, 80%, and 20% of the maximum dose, respectively.

The beam validation experiments were modelled in MCNP 6.3 as a monoenergetic proton beam with radius 0.25 cm directed at the water tank. To calculate dose deposition, a TMESH type 3 tally was used, covering the tank and recording the average energy deposition per unit volume (MeV cm^-3^ per proton history) at 500 points in the direction of the beamline. The mesh tally voxel dimension was 2.0 cm × 2.0 cm × 0.1 cm. A data analysis based on a Bortfeld fit (38) was then carried out to determine the simulated values of R90, R80, and R20. A schematic of the modelling is shown in **Figure 4**.

#### 2.2.3 Influence of room walls on the neutron spectra

To highlight the importance of including the bunker geometry, simulations of a 210 MeV proton beam incident on a Solid Water phantom were performed with and without the bunker for Case A. Neutron fluence energy spectra at the BSS position were calculated using a F4 tally in both cases. The average and maximum difference between the calculated spectra are then evaluated in section 3.3.

### 2.3 Spectra unfolding

A previously developed (35, 39) Maximum Likelihood Expectation Maximization (MLEM) unfolding algorithm was used to unfold the neutron spectra for Cases A, B, and C. The method assumes that the neutron counts in the detector are related to the neutron fluence energy spectrum as follows:


$$m_{i}=\int_{E_{min}}^{E_{max}}R_{i}(E)n(E)dE,$$

where $m_{i}$ are the counts in the detector for the *i*-th BSS sphere, $R_{i}(E)$ is the detector energy response function and n(E) is the unknown fluence energy spectrum. The MLEM algorithm iterates until converging on the neutron spectrum with the measurements. In this work, a BSS response matrix from previous studies (35) was adapted to incorporate the response of the extended-range spheres, allowing determination of the neutron spectrum up to 250 MeV. As an initial guess, the MLEM method uses a spectrum predicted by the MC simulations. The neutron counts given to the MLEM algorithm are formed by the experimental measurements recorded for spheres 1 to 6 at MPTC and the MC simulated counts for spheres 7 to 10. To ensure all the neutron counts in the set were on the same scale, the average proportional factor between experimental and MC-simulated counts was calculated. The resulting factors for Cases A, B, and C were 3.45 × 10^3^, 1.62 × 10^5^, and 2.50 × 10^5^ experimental counts per simulated count, normalized per emitted particle. The simulated counts were then multiplied by the corresponding factor prior to unfolding the spectra.

## 3 Results

### 3.1 Methodology validation

**Figure 5:**
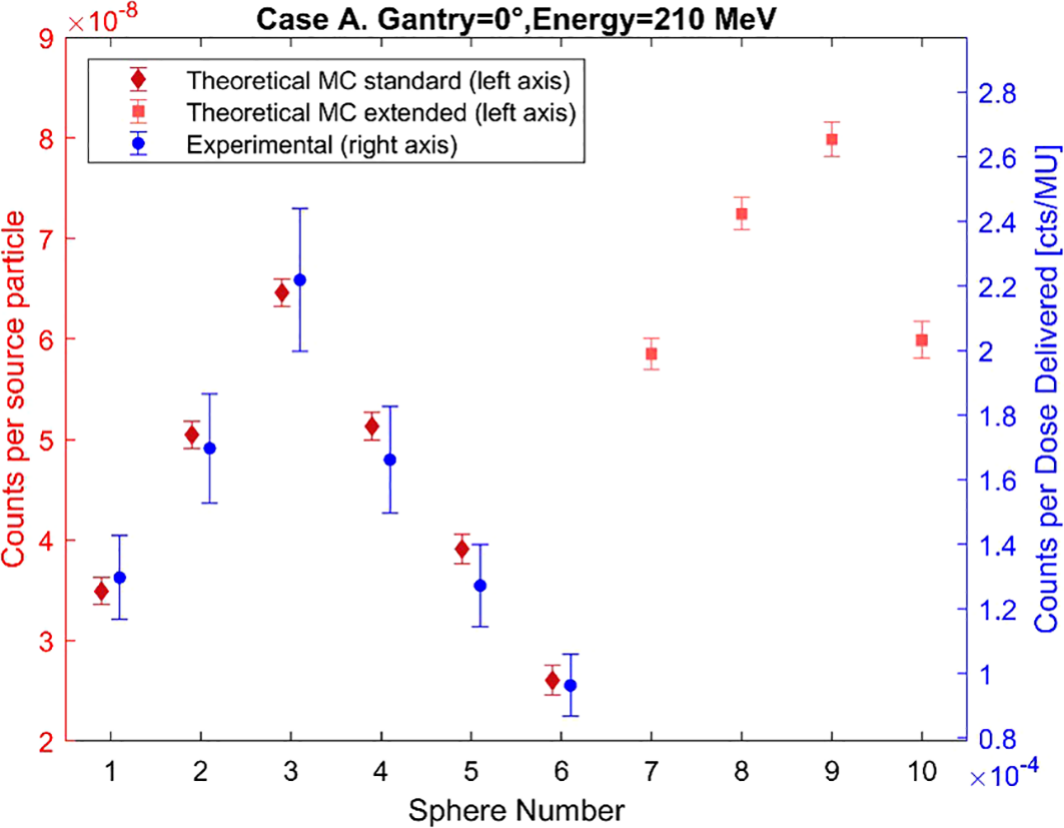
Comparison of the simulated and experimental neutron counts registered by the BSS for Case A. The data points have been slightly horizontally offset to prevent overlap.

**Figure 6:**
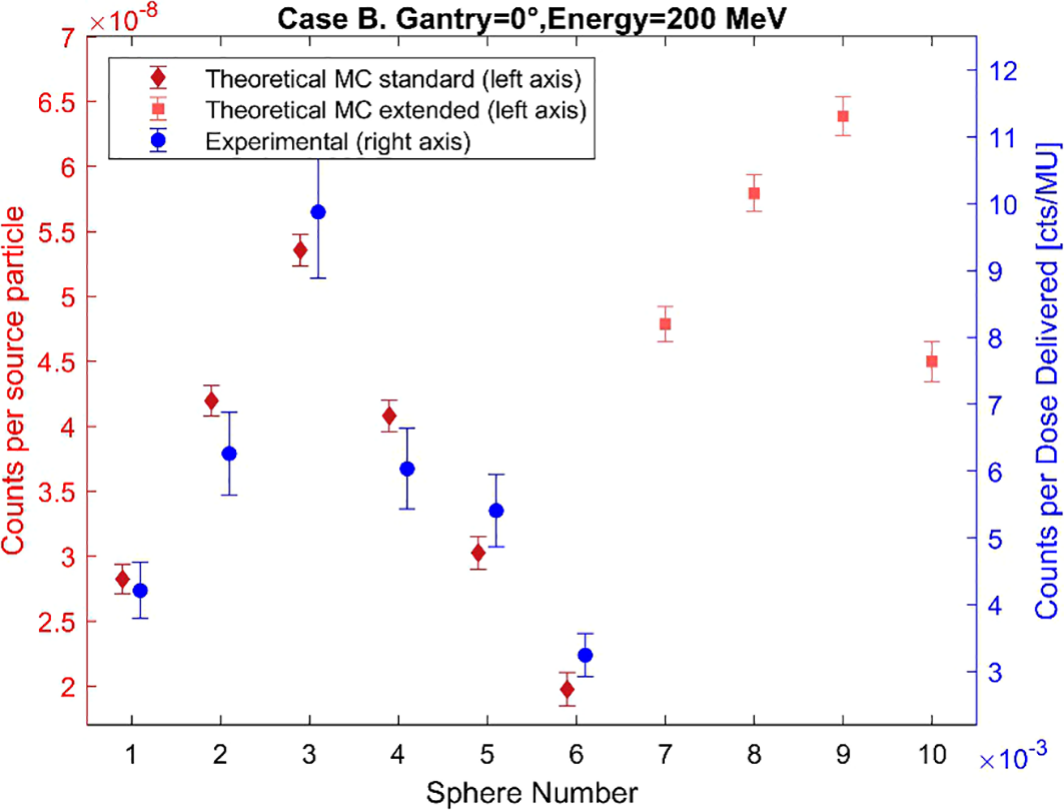
Comparison of the simulated and experimental neutron counts registered by the BSS for Case B. The data points have been slightly horizontally offset to prevent overlap.

**Figure 7:**
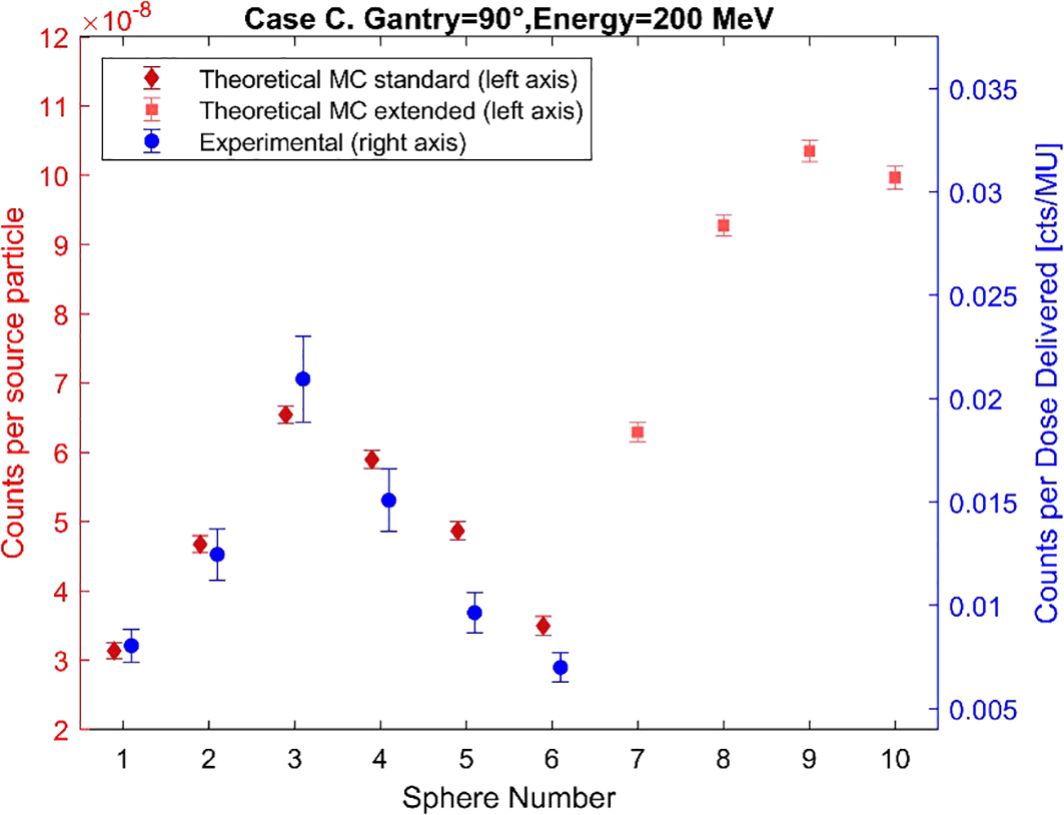
Comparison of the simulated and experimental neutron counts registered by the BSS for Case C. The data points have been slightly horizontally offset to prevent overlap.

To validate the MC model of the standard BSS, the simulated and experimental counts for each of the BSS spheres have been plotted for Cases A, B, and C in **Figures 5**–**7**, respectively. The MC count values for the extended-range spheres are also shown in **Figures 5**–**7**. The simulated results reproduce the same trend as the experimental measurements within the estimated uncertainty for most of the spheres, with the maximum response recorded by sphere 3, but sphere 9 has the maximum response in all cases when considering the extended-range spheres. The median, average, minimum, and maximum differences are 15.4%, 14.9%, 1.0%, and 36.8%. The simulated counts increase on average by 25% from Case B to Case A and by 50% from Case B to Case C.

### 3.2 Spectra unfolding

**Figure 8:**
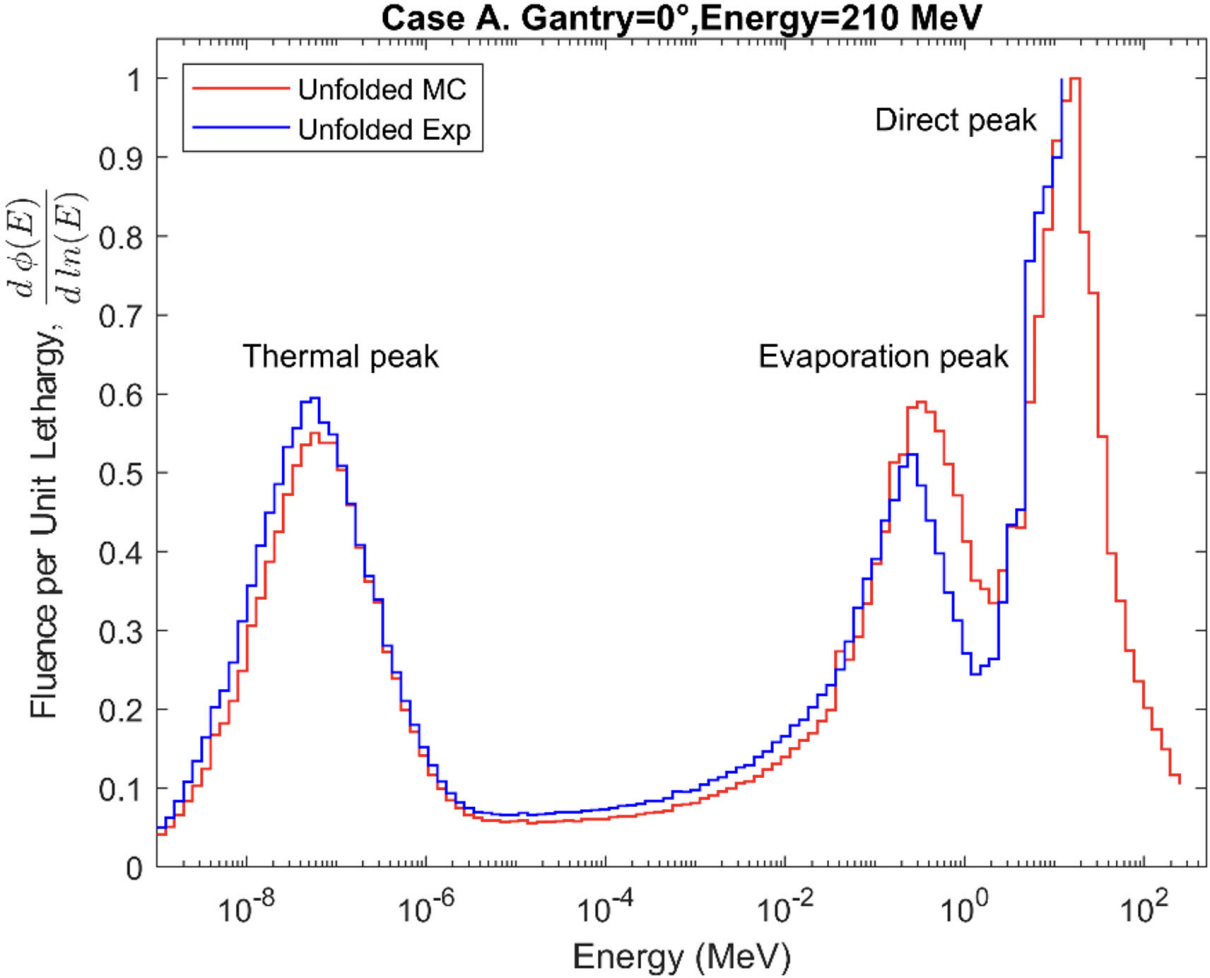
Secondary neutron energy fluence spectra for Case A.

**Figure 9:**
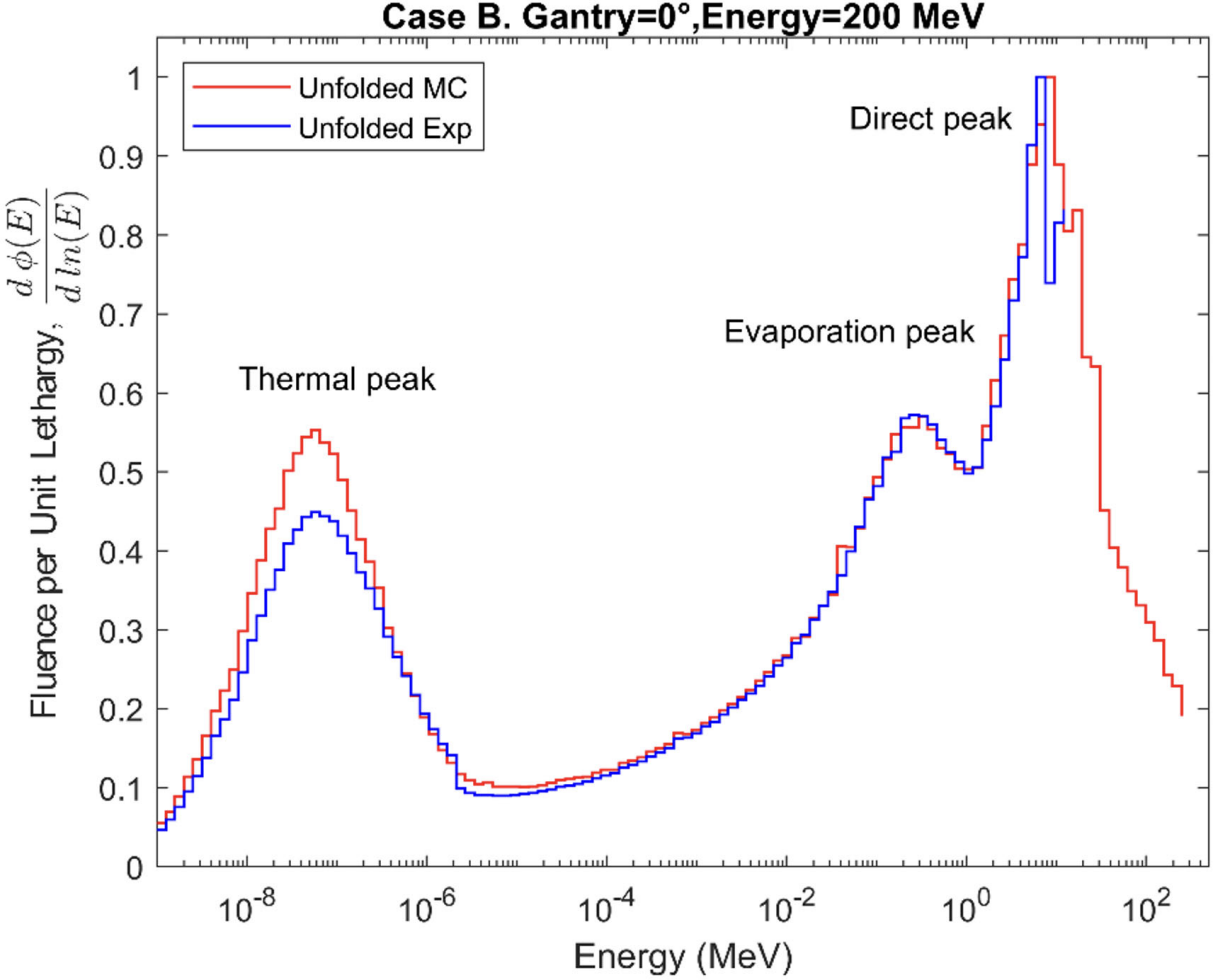
Secondary neutron energy fluence spectra for Case B.

**Figure 10:**
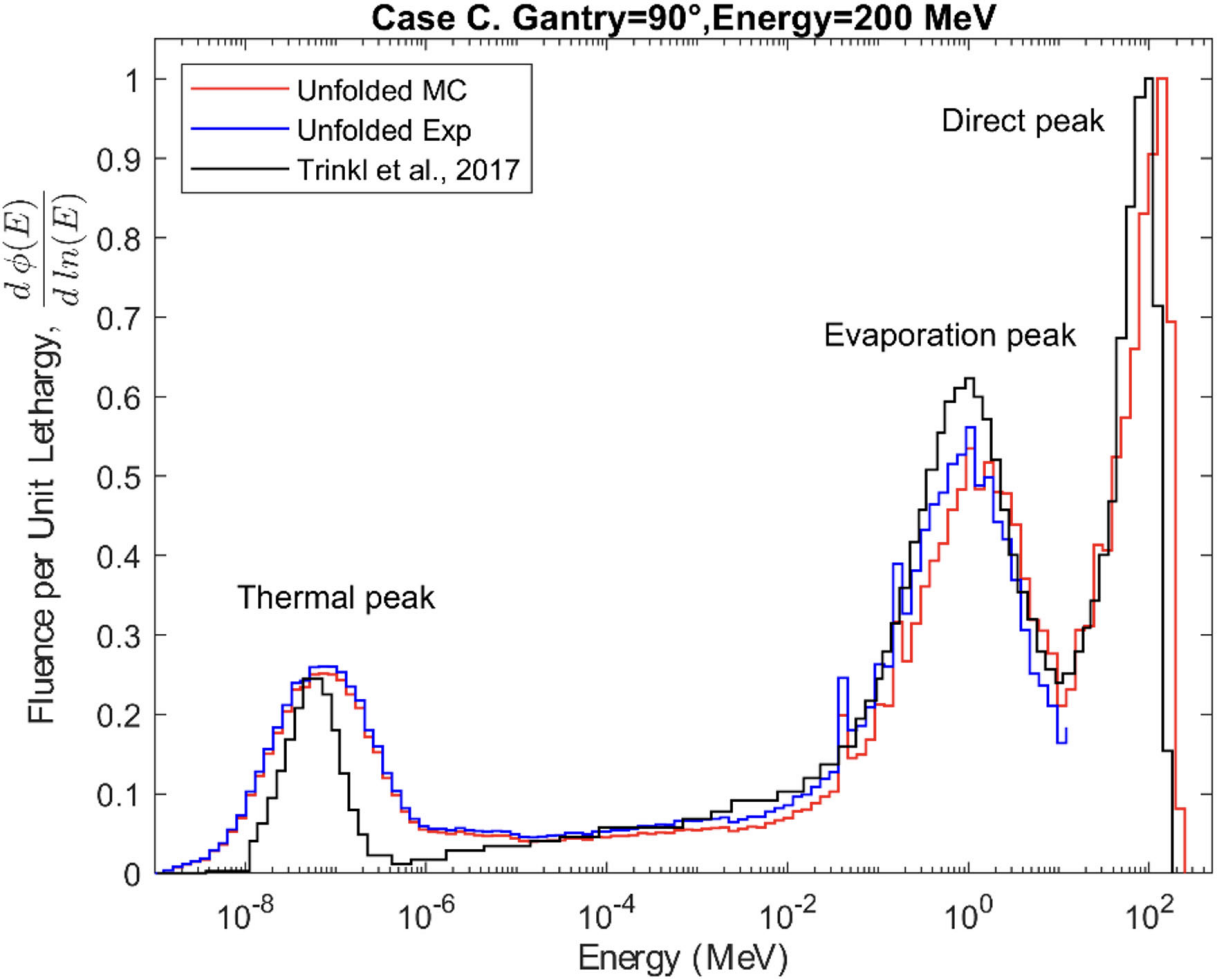
Secondary neutron energy fluence spectra for Case C. The experimental measurements were compared both with the simulations obtained using our model and with those derived from a similar model presented in the study by Trinkl et al. (13).

The neutron fluence energy spectra were unfolded using the MLEM algorithm and normalized per unit lethargy as a function of neutron energy. The results for Cases A, B, and C are shown in **Figures 8**–**10**, respectively. The figures compare the spectra unfolded exclusively from the MC simulated counts to the spectra unfolded from the experimental counts for spheres 1 to 6. In Cases A, B, and C the average differences between both spectra up to 12 MeV are 6.9%, 6.4%, and 8.6%. The numerical values of the positions and intensities of the peaks that make up the MC spectra for the three cases are shown in **Table 1**. For Case A, the direct peak and the evaporation peak (E > 0.1 MeV) are more intense compared to the thermal and moderation regions (E < 0.1 MeV), with the direct peak being 82% more intense than thermal peak. In Case B an 84% broader evaporation peak and a 20% higher relative intensity in the moderation region are observed. Finally, a less intense thermal peak can be seen in Case C while the overall shape of the spectrum is consistent with the previous cases.

**Table 1:** Energy and intensity of the thermal and evaporation peaks relative to the direct peak for the three experimental cases (Cases A, B, and C).

	Thermal peak		Evaporation peak		Direct peak	
Case	E (MeV) ·10^-8^	Rel. intensity	E (MeV)	Rel. intensity	E (MeV)	Rel. intensity
Case A	5.18	0.55	0.30	0.59	15.36	1
Case B	5.18	0.40	0.30	0.57	7.63	1
Case C	6.53	0.25	0.94	0.54	123.72	1

### 3.3 Influence of room walls on the neutron spectra

**Figure 11:**
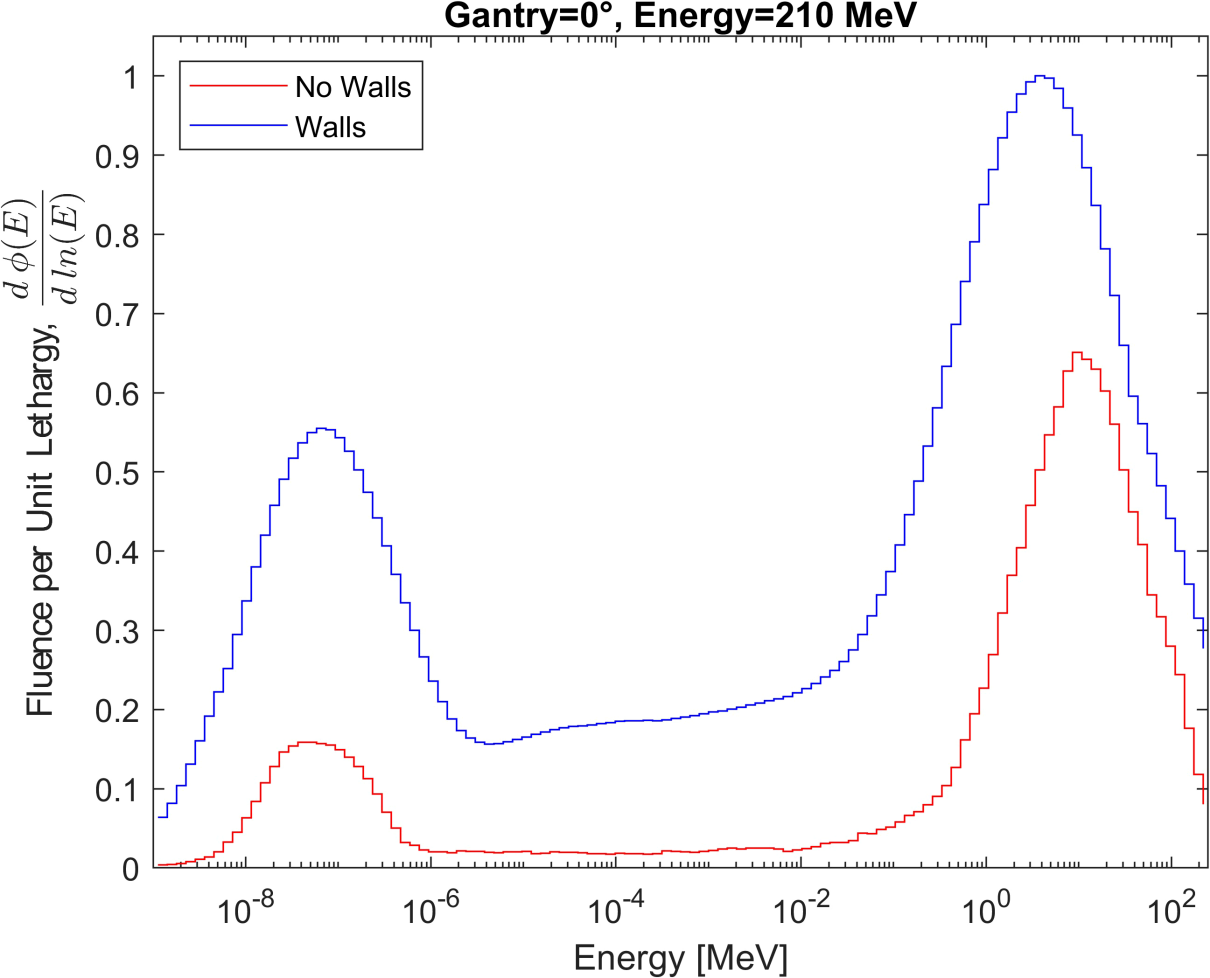
Comparison of the secondary neutron fluence spectra with and without considered bunker geometry for Case A.

The effect of bunker walls on secondary neutron spectra was evaluated by simulating the spectra at the position of Case A with and without bunker walls. **Figure 11** presents the spectra normalized per unit lethargy as a function of neutron energy. Both spectra were smoothed by the average of adjacent bins to remove any artificial peaks in the unfolding spectra. This resulted in a broad peak that combined the direct and evaporation peaks, as previously reported by Trinkl et al. (13) and Mares et al. (40). When the bunker is included, the relative intensity of the thermal peak (10^-9^ MeV to 10^-6^ MeV) compared to the broad peak (a few MeV) increased by a factor of 2.3. The contribution of moderation neutrons (1 eV to 0.1 MeV) with the bunker is 8.8 times higher. The total fluence over the entire energy range was calculated to be 3.795×10^-6^ neutrons·cm^-2^ without the walls and 6.722×10^-6^ neutrons·cm^-2^ when they were included. Including the bunker shifts the location of the broad peak lower by 5 MeV. The spectra differ for Case A on average by 77%, with a maximum ratio of 11.7 observed. The effect has also been studied at the position of Case C, obtaining an average difference of 48% between both spectra, with a maximum difference of 2.0. Regarding the fluence, an increase of 68% is observed with respect to the position of Case A.

### 3.4 Beam validation

**Figure 12:**
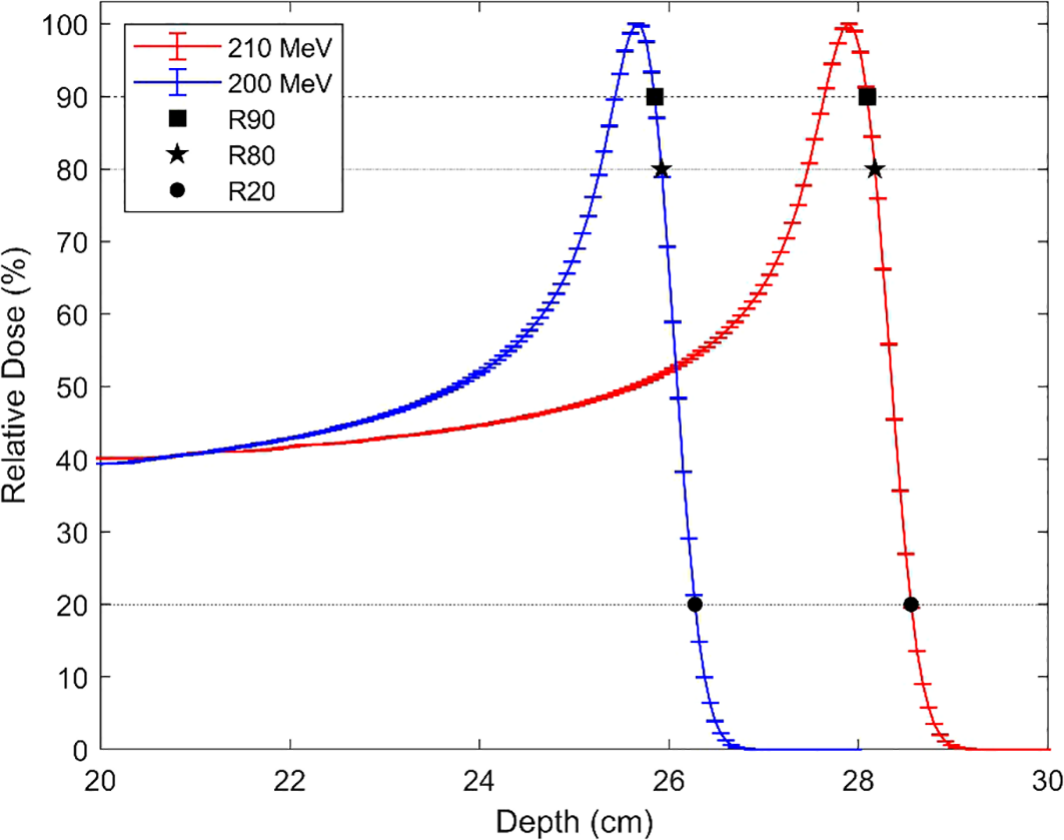
MC calculated depth dose curves for 200 MeV and 210 MeV protons showing the positions of R90, R80, and R20.

**Figure 13:**
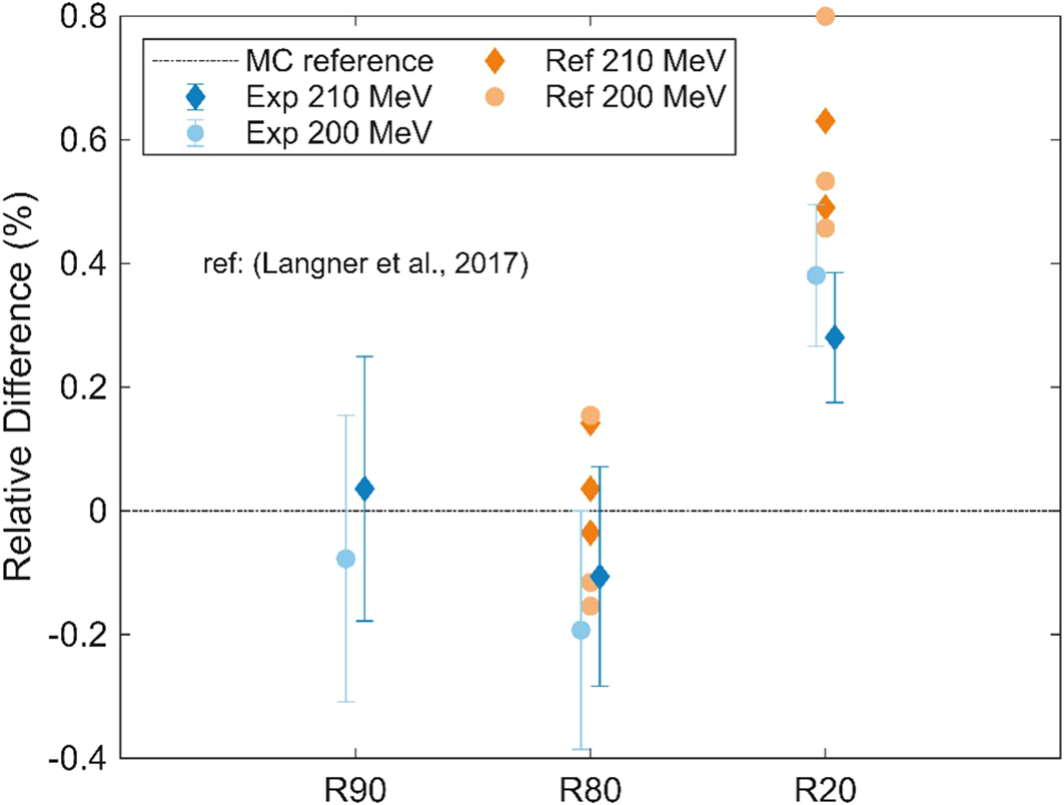
Relative difference between MC, experimental, and reference (26) measurements of R90, R80, and R20. The experimental data points have been slightly offset horizontally to prevent overlap.

The depth-dose curves for 200 MeV and 210 MeV protons were calculated and are shown in **Figure 12**. The Monte Carlo statistical uncertainties for each point of the depth-dose curve were lower than 1.5%. The calculated MC and experimental positions of R90, R80, and R20 are compared in **Table 2**. For the simulated data, the R90, R80, and R20 at 200 MeV and 210 MeV differ by 2.25 cm, 2.25 cm, and 2.28 cm, respectively. For the three experimental measurement points, the depth difference is 2.27 cm. These results yield an average difference of 2.26 cm and of 2.27 cm for experimental and simulated, respectively, with an average relative difference of 0.44%. The relative difference between MC and experimental measurements for the R90, R80, and R20 were below 0.38%, with the 210 MeV dataset showing the smallest difference. A comparison with prior published measurements by Langner et al. (26) is shown in **Figure 13**, reporting differences below 0.8% in all cases.

**Table 2:** Comparison of simulated (MC) and experimental measurements (EXP) of R90, R80, and R20.

Energy (MeV)	R90 Depth (cm)			R80 Depth (cm)			R20 Depth (cm)		
	MC	EXP	Diff (%)	MC	EXP	Diff (%)	MC	EXP	Diff (%)
200	25.85	25.83 ± 0.06	0.08	25.92	25.87 ± 0.05	0.19	26.27	26.37 ± 0.03	0.38
210	28.08	28.10 ± 0.06	0.04	28.17	28.14 ± 0.05	0.11	28.56	28.64 ± 0.03	0.28

## 4 Discussion and conclusions

The primary goal of this work was to establish a robust methodology for neutron spectrum reconstruction based on six experimental measurements, applicable across both medical and non-medical facilities. Although developed within the context of medical proton therapy using a Solid Water block to simulate a patient, the approach is transferable to other environments where neutrons with energies up to 250 MeV are present. Examples include particle accelerators, where high-energy neutrons up to hundreds of MeV are generated (41).

The simulated beam model was compared with experimental measurements and previously published data, showing agreement and confirming that it accurately reproduces the proton Bragg peak. This suggests reliability in the subsequent neutron generation; however, the number of produced neutrons also depends on the chosen Monte Carlo code, underlying physical models, and nuclear data libraries.

The designed BSS detection model captures changes in beam and measurement conditions, as evidenced by consistency in data trends with the experimental measurement counts with the conventional BSS in the three cases studied. Case A and B share the same beam orientation, but slightly higher energy and closer proximity to the water tank in Case A produces higher counts than in Case B. Case C shows the highest counts due to the smaller angle between the beam and the measurement position, which increases the number of neutrons reaching the detector. Case C shows the largest difference between the experimental and MC counts, specifically for spheres 5 and 6. These differences can be associated with various sources of uncertainty, namely a discrepancy in the detector’s position during measurement, a possible underestimation of the experimental results, room components not included in the model that may affect the number of simulated neutrons reaching the detector, and uncertainties related to the selected cross sections and neutron physics models.

Although experimental data for the extended-range BSS is unavailable, our simulations adapted a BSS response matrix from previous studies (35) to include the response of the extended-range spheres, allowing determination of the neutron spectrum up to 250 MeV. The simulated counts for the extended-range spheres properly predict the higher neutron counts relative to the conventional BSS, where spallation reactions of high-energy neutrons result in increased moderation and detection (15).

All the obtained spectra show the characteristic shape of the secondary neutron spectrum indicated in previous works (11–13), with system−specific variations in the balance between fast and evaporation neutrons. The differences between the shapes of the spectra from MC counts and experimental data, observed in **Figures 8**–**10**, remain below 9% in all cases, thereby confirming the validation of the MC model up to 12 MeV. These differences can also be explained by the differences in the recorded counts shown in **Figures 5**–**7**, together with the other possible uncertainties listed above. The variations in neutron spectra between different cases reflect the combined effects of beam energy, detector placement, and room geometry. In Case A, the 90° off-axis position shifts the direct peak toward lower energies, while the higher beam energy increases the intensity of this peak. The relatively higher direct peak with respect to the evaporation peak can be associated with a possible underestimation of evaporation neutrons, which may arise from the simplified room model that does not include all scattering surfaces. In Case B, proximity to the walls enhances neutron reflections, increasing the contribution of evaporation and moderation neutrons, while the off-axis placement continues to limit the detection of direct high-energy neutrons and shift the peak to lower energy. In Case C, moving the detector closer to the beamline increases the proportion of direct neutrons, shifting the direct peak to higher energy. The spectrum in Case C is consistent with previous measurements by Trinkl et al. (13) in a similar case of study, supporting the applicability of the model up to 12 MeV. The measurement conditions in both cases are very similar, differing slightly in the radial distance between the phantom and the detector, hence providing support for the validity of the model under similar experimental conditions. To our knowledge there are no universally accepted radiation protection performance criteria that define accuracy requirements for spectral shape reconstruction in proton therapy environments. Performance expectations are typically framed in terms of agreement of derived dose-equivalent quantities such as H*(10) rather than spectral shape, reflecting the intrinsic challenges associated with measuring and modeling broad, high-energy neutron fields.

The analysis on the effect of bunker walls showed that including the bunker geometry increases the contributions of thermal and moderation neutrons, indicating that neglecting room geometry could lead to their underestimation on average by 77% (Case A) or 48% (Case C). These findings confirm that incorporating bunker geometry in simulations is essential for a complete description of secondary neutron production, in agreement with prior studies (13, 40).

Although the present study provides robust results, certain aspects could be further refined. We also recognize a key limitation of the BSS is absence of a single solution for deconvoluted spectra, as the results represent the closest approximations to the real spectra obtained when multiple response functions converge. Future efforts should focus on three key areas. First, uncertainties in neutron spectra would be reduced by implementing a more detailed room model that includes all relevant scattering surfaces and equipment, as the model used includes only the walls of the bunker. The contribution of individual room elements could even be studied, as done by 32. Second, manufacturing extended-range spheres and performing targeted experimental measurements would validate the high-energy neutron simulations and strengthen confidence in high-energy neutron predictions above 12 MeV. Finally, additional neutron spectrum measurements at more proton energies and angles, as well as using paired gold foils to assess the absolute thermal region of the spectra, would enhance model reliability and broaden its applicability to a wider range of clinical conditions.

## Data Availability

The original contributions presented in the study are included in the article/supplementary material. Further inquiries can be directed to the corresponding author.
